# Antimicrobial and Apoptotic Efficacy of Plant-Mediated Silver Nanoparticles

**DOI:** 10.3390/molecules28145519

**Published:** 2023-07-19

**Authors:** Elżbieta Radzikowska-Büchner, Wojciech Flieger, Sylwia Pasieczna-Patkowska, Wojciech Franus, Rafał Panek, Izabela Korona-Głowniak, Katarzyna Suśniak, Barbara Rajtar, Łukasz Świątek, Natalia Żuk, Anna Bogucka-Kocka, Anna Makuch-Kocka, Ryszard Maciejewski, Jolanta Flieger

**Affiliations:** 1 Department of Plastic, Reconstructive and Maxillary Surgery, CSK MSWiA, 02-507 Warszawa, Poland; 2 Department of Anatomy, Medical University of Lublin, 20-090 Lublin, Poland; wwoj24@gmail.com (W.F.); ryszard.maciejewski@umlub.pl (R.M.); 3 Department of Chemical Technology, Faculty of Chemistry, Maria Curie Skłodowska University, Pl. Maria Curie-Skłodowskiej 3, 20-031 Lublin, Poland; sylwia.pasieczna-patkowska@mail.umcs.pl; 4 Department of Geotechnics, Civil Engineering and Architecture Faculty, Lublin University of Technology, Nadbystrzycka 40, 20-618 Lublin, Poland; w.franus@pollub.pl (W.F.); rapanek@gmail.com (R.P.); 5 Department of Pharmaceutical Microbiology, Medical University of Lublin, Chodźki 1 St., 20-093 Lublin, Poland; izabela.korona-glowniak@umlub.pl (I.K.-G.); katarzyna.susniak@umlub.pl (K.S.); 6 Department of Virology with Viral Diagnostics Laboratory, Medical University of Lublin, Chodźki 1, 20-093 Lublin, Poland; barbara.rajtar@umlub.pl (B.R.); lukasz.swiatek@umlub.pl (Ł.Ś.); 7 Department of Analytical Chemistry, Medical University of Lublin, Chodźki 4A, 20-093 Lublin, Poland; natalia.zuk@umlub.pl; 8 Chair and Department of Biology and Genetics, Medical University of Lublin, 4a Chodźki St., 20-093 Lublin, Poland; anna.bogucka-kocka@umlub.pl; 9 Department of Pharmacology, Medical University of Lublin, 4a Chodźki St., 20-093 Lublin, Poland; anna.makuch@umlub.pl

**Keywords:** silver nanoparticles, green synthesis, *Tanacetum vulgare* L. extracts, bioactive phytochemicals, antibacterial activity, anticancer activity

## Abstract

Phytogenically synthesised nanoparticle (NP)-based drug delivery systems have promising potential in the field of biopharmaceuticals. From the point of view of biomedical applications, such systems offer the small size, high surface area, and possible synergistic effects of NPs with embedded biomolecules. This article describes the synthesis of silver nanoparticles (Ag-NPs) using extracts from the flowers and leaves of tansy (*Tanacetum vulgare* L.), which is known as a remedy for many health problems, including cancer. The reducing power of the extracts was confirmed by total phenolic and flavonoid content and antioxidant tests. The Ag-NPs were characterised by various analytical techniques including UV–vis spectroscopy, scanning electron microscopy (SEM), energy-dispersive spectrometry (EDS), Fourier transform infrared (FT-IR) spectroscopy, and a dynamic light scattering (DLS) system. The obtained Ag-NPs showed higher cytotoxic activity than the initial extracts against both human cervical cancer cell lines HeLa (ATCC CCL-2) and human melanoma cell lines A375 and SK-MEL-3 by MTT assay. However, the high toxicity to Vero cell culture (ATCC CCL-81) and human fibroblast cell line WS-1 rules out the possibility of their use as anticancer agents. The plant-mediated Ag-NPs were mostly bactericidal against tested strains with MBC/MIC index ≤4. Antifungal bioactivity (*C. albicans, C. glabrata,* and *C. parapsilosis*) was not observed for aqueous extracts (MIC > 8000 mg L^−1^), but Ag-NPs synthesised using both the flowers and leaves of tansy were very potent against *Candida* spp., with MIC 15.6 and 7.8 µg mL^−1^, respectively.

## 1. Introduction

Noble metal nanoparticles (NPs) such as gold (Au), silver (Ag), platinum (Pt), and copper (Cu) have found many industrial applications in catalysis and electronics [1,2,3,4,5]. Recent studies have focused on their use in biomedicine [6,7] as carriers of targeted drugs [8,9,10] and as biosensors for medical diagnostics [11,12,13,14,15,16,17,18]. The unique physicochemical properties of NPs result from, among other things, the size-dependent surface plasmon response the quantum confinement effect and large reactive surfaces [19,20,21].

Ag has been valued as a precious metal for thousands of years. Hippocrates and the Phoenicians were aware of the antiseptic properties of silver. In the 19th century, silver compounds were used to prevent the transmission of gonorrhoea from mother to newborn, and during World War I, they were used to prevent infection [22]. The antipathogenic effect of Ag-NPs has been shown to be superior to that of Ag ions [23,24]. The high antimicrobial efficacy and nontoxicity of Ag-NPs is now widely used in wound dressings, tissue scaffolds, and protective clothing applications [25]. The antimicrobial activity of Ag-NPs has been experimentally confirmed against pathogenic microorganisms such as bacteria [26,27,28], viruses [29,30], fungi, and yeasts [31,32]. Although the exact antipathogenic mechanism of Ag-NPs remains to be elucidated [33], it is known that it depends on various NP characteristics, i.e., particle shape, particle size, concentration, surface charge, surface modification, and colloidal state [34,35,36,37,38,39,40]. An alternative to Ag-NP is the commercial Tegaderm™ foil dressing or a recently developed hydrogel synthesised by repeated freeze–thaw, consisting of poly(vinyl alcohol) (PVA), silk sericin (SS), and azithromycin (AZM), with genipin (GNP) as a cross-linking agent [41]. In this dressing, the antimicrobial effect is mainly due to the addition of the AZM antibiotic. Ag-NPs have also been tested for their anticancer activity on various cancer cell lines such as endothelial cells, IMR-90 lung fibroblasts, U251 glioma cells, and MDA-MB-231 breast cancer cells [42,43]. In biomedical applications, the nontoxic and nonimmunogenic nature of NPs should be ensured. In general, Ag-NPs are considered to be harmless to mammalian cells [44]. However, studies on the toxic effects of Ag-NPs in different biological systems have shown inconsistent results [45,46]. Some animal studies have shown toxic effects of Ag-NPs on rat hepatocytes and neurons [47], mouse stem cells, and human lung epithelial cells [48]. It should be emphasised that both in vitro and in vivo toxicity of Ag-NPs is dependent on exposure time and dose [49].

NPs obtained by chemical synthesis not only cause environmental pollution due to the toxic reagents used in the reaction [50], but also pose a threat to humans due to the cytotoxicity of the NPs produced [51]. The green synthesis of NPs, which is environmentally friendly, offers the possibility of obtaining high-quality monodisperse NP systems with controlled size and shape [52,53]. NPs obtained by green synthesis methods use natural extracts or living organisms as a source of chemicals, i.e., flavonoids, alkaloids, phenols, terpenoids, proteins, sugars, and coenzymes, which act as reducers and stabilisers of NPs [54,55]. To ensure the synthesis and functionalisation of nanobiological constructs, a high level of antioxidants is required in the reaction mixture. The modification of the surface properties of NPs by biomolecules conjugated to NPs affects the diversified biological activity [56,57,58,59].

In this study, monodisperse colloidal Ag-NPs were synthesised by green synthesis using plant extracts of *Tanacetum vulgare* L. belonging to the *Asteraceae* family. The *Asteraceae* family contains about 25,000 species. More than 300 species have been identified in Poland. Plants from this family contain various bioactive compounds, e.g., essential oils [60,61,62,63], polyphenolic compounds [64,65], flavonoids [64,65,66,67,68,69,70], terpenoids [67,68,69,71,72,73,74,75,76], phenolic acids [66,69,77], alkaloids [78], lignans [66], saponins [67,74], stilbenes, sterols [69], polysaccharides [71], and others. The medicinal properties of plants in the *Asteraceae* family are used in folk medicine for many health problems, including cancer [79]. An example is *Artemisia absinthium* L. (wormwood), which has insecticidal, anthelmintic [60,72], antimalarial [73,80], antiseptic, anti-inflammatory, and antioxidant properties [81]. *Arctium lappa* L. (burdock) is used as an anti-aging, antioxidant, antimutagenic, anticancer, and anti-inflammatory agent [66,82]. *Calendula officinalis* L., best known as a remedy for skin eruptions and abrasions [74,75,83], has also been shown to have cytotoxic, anticancer [77], and anti-HIV effects [68]. *Tragopogon pratensis* L. has diaphoretic and antitussive properties [84]. *Tanacetum vulgare* L. (tansy) is an aromatic perennial plant native to Eurasia. Tansy contains essential oils [85,86,87,88,89], nonvolatile compounds (e.g., lactones), and phenolic compounds (e.g., phenolic acids, flavonoids) [90,91,92,93]. In animal studies (male Wistar rats), an aqueous extract of tansy leaves has been shown to be nontoxic to the kidneys and to show no other side effects [94]. The content of active substances in the plant is most influenced by the anthropogenic environment [95,96,97,98], which is affected by animal husbandry [99], industry (pharmaceutical and medical [100,101], food, chemical and petroleum [102,103,104,105], etc.), and wastewater [106,107,108,109,110]. Another factor is weather conditions, which affect the content of active ingredients in each plant [90,91,92,95]. Tansy extracts showed antibacterial properties in a study with *Escherichia coli* and *Staphylococcus aureus* [111], as well as antifungal and repellent properties [111,112,113,114]. *Tanacetum vulgare* L. is used in traditional medicine to treat many health problems such as digestive disorders, rheumatism, fever, and ulcers [71]. Anti-inflammatory [70,71,115] and anticancer activities [115] have also been reported.

In this study, extracts of different aerial parts of *Tanacetum vulgare* L. (flowers and leaves) collected in eastern Poland were prepared with water or 80% ethanol. In the first step, the antioxidant activity, phenolic acid, and total phenolic content of the extracts were determined. They were then used as reducing and stabilising agents in the green synthesis of Ag-NPs. The synthesised green Ag-NPs were evaluated for their antimicrobial activity and, for the first time, for their anticancer activity against human cervical cancer cell lines and human melanoma cell lines in comparison to pure extracts.

## 2. Results

### 2.1. Antioxidant Capacity of Extracts Evaluated Using SNPAC and FRAP Tests

Different reaction mechanisms, i.e., hydrogen transfer (HAT), electron transfer (ET), and mixed mode (HAT/SET), were used in the tests to measure antioxidant activity [116]. Since the suitability of plant extracts for the synthesis of NPs mainly depends on their reducing capacity, the most suitable tests for the evaluation of antioxidant capacity were those offering the ET mechanism, i.e., the reduction of the antioxidant power of iron (FRAP) and the test of antioxidant capacity of the SNPAC (silver nanoparticle antioxidant capacity) assay [117]. Since the prepared extracts are rich in different antioxidants, thanks to which they become electron donors in the formation of metallic nanoparticles, the suitability of extracts for this purpose can also be evaluated on the basis of the total phenolic content using the Folin–Ciocalteu (FC) method and the total flavonoid content (TFC) method [118]. The potential of plant extracts to reduce silver ions was evaluated separately for *Tanacetum vulgare* extracts obtained from flowers (F) or leaves (L) and different types of solvents used for extraction (I-water, II-80% ethanol).

#### 2.1.1. SNPAC

**Figure 1:**
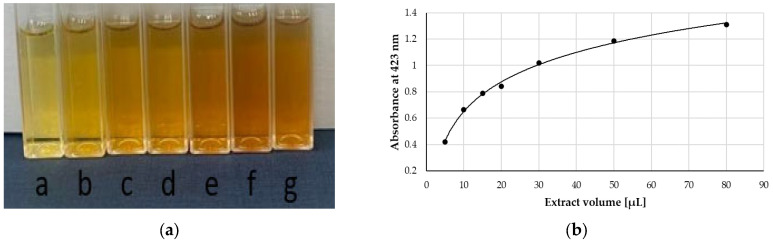
(**a**) A colour image of the test tubes containing Ag-NPs formed in the presence of different volumes of *Tanaceti flos* aqueous extract: (a) 5 µL, (b) 10 µL, (c) 15 µL, (d) 20 µL, (e) 30 µL, (f) 50 µL, and (g) 80 µL. (**b**) Absorbance measured at 423 nm versus the volume of extract added.

The SNPAC method was used to evaluate the total antioxidant capacity (TAC) of the extracts studied. The test samples for the standard calibration curve were prepared by mixing Ag-NPs with the standard and water. After storage in the dark, the mixtures turned dark brown (Figure 1a). The standard calibration curve represents the relationship between the absorbance at the wavelength of 423 nm and the final molar concentration of Trolox (TR) used as a reference antioxidant (Figure 1b). The calibration curve obtained was linear in the concentration range from 3.57 to 71.40 µM. The equation for the calibration curve was as follows:

3014.165 (±89.73)x + 0.2143 (±0.0039), R^2^ = 0.9973, F = 1128.39, se = 0.026 (1)

The LOD for TR was found to be 3.88 μM, calculated using the equation; LOD = 3 sd/s, where sd is the standard deviation of a blank and s is the slope of the calibration curve. The obtained LOD value is higher compared to the value of 0.23 μM obtained by Özyürek et al. [117], but their concentration range was much wider within 1.28 × 10^−7^–1.12 × 10^−4^ M. The total antioxidant capacity (TAC) of the extracts was calculated by dividing the observed absorbance at λ = 423 nm by the molar absorptivity (ε) of TR, which can be obtained from the slope of the calibration curve.

**Figure 2:**
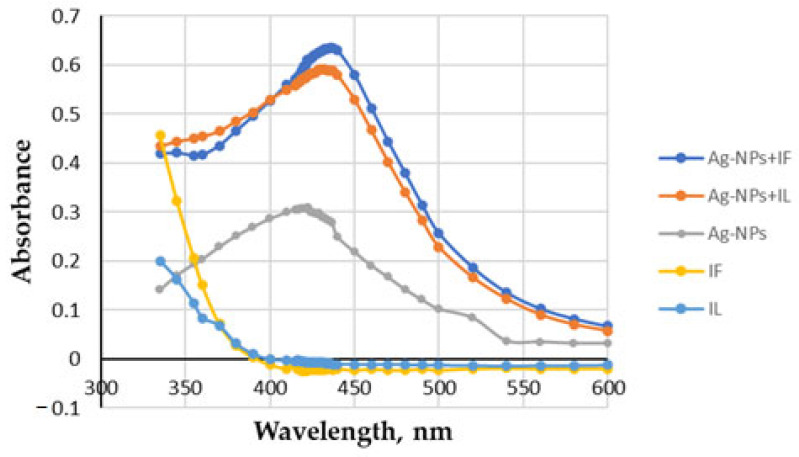
UV−vis spectra of the *Tanaceti flos* (IF) and *Tanaceti folium* (IL) aqueous extracts, initial Ag-NPs, and Ag-NPs formed in the presence of extracts (Ag-NPs + IF, Ag-NPs + IL).

Figure 2 shows the absorption spectra of the extracts and the initial solution of Ag-NPs with the citrate capping agent, and the changes observed after mixing the two solutions, prepared separately for aqueous extracts of flowers and leaves of *Tanacetum vulgare* L. As can be seen, the extracts alone give a significant absorbance at 423 nm, but after the addition of the Ag-NPs solution, an increase in the absorption band around 420 nm is observed. This band is characteristic of the surface plasmon resonance of Ag-NPs [119].

The reducing potential of the tansy extracts could be observed by the colour change. After adding the initial Ag-NPs solution to the extract and incubating for 30 min, the colour changed from yellow or green to dark brown. The UV–vis spectrum of the extract was also characterised by a broad band with a maximum below 350 nm, indicating a high content of polyphenolic compounds [120,121].

#### 2.1.2. FRAP

The FRAP method is based on the ability of antioxidants to reduce Fe^+3^ to Fe^+2^ in the presence of TPTZ, forming an intense blue Fe^+2^–TPTZ complex. The absorbance of the extracts was compared with an ascorbic acid (vitamin C) standard curve, and FRAP values were expressed as vitamin C equivalents per volume of extract. The calibration curve was linear over the concentration range from 6.25 to 100.0 µg mL^−1^. The equation for the calibration curve was as follows:

8.917 (±0.247)x − 0.0215 (±0.012), R^2^ = 0.9977, F = 1300.376, se = 0.462 (2)

The LOD for ascorbic acid was determined to be 1.35 μg/mL using the equation: LOD = 3 sd/s, where sd is the standard deviation of a blank and s is the slope of the calibration curve.

#### 2.1.3. DPPH

The IC50 values at which the sample reduces 50% of the absorbance intensity of DPPH free radicals were converted to the concentration of chlorogenic acid (CGA) used as a reference antioxidant (mg mL^−1^ extract). The CGA calibration curve was linear over the concentration range 1.77 to 10.62 µg mL^−1^. The equation for the calibration curve was as follows:

2940.65(±95.97)x − 1.269 (±1.479), R^2^ = 0.9978, F = 938.942, se = 1.730 (3)

The LOD for chlorogenic acid was determined to be 0.535 μg mL^−1^ using the equation: LOD = 3 sd/s, where sd is the standard deviation of a blank and s is the slope of the calibration curve.

Analysis of the extracts using the SNAPC, FRAP, and DPPH methods showed that the antioxidant activity was highest in hydroalcoholic extracts compared to aqueous extracts. Furthermore, flowers showed greater antioxidant activity than leaves in the tests used (Table 1). Statistical analysis of the data collected using Student’s t-test confirmed the existence of significant differences (*p* ≤ 0.05) in the antioxidant activity of the extracts.

#### 2.1.4. Total Phenolic and Flavonoid Content

The determination of polyphenolic content is important because these compounds can contribute as reducing agents in the synthesis of Ag-NPs, providing the necessary electrons for the reduction of silver ions to metallic silver [122]. Polyphenolic compounds and flavonoids are not only responsible for the antioxidant capacity of the extracts but also for the stabilisation of the NPs, ensuring good surface coverage and dispersion of the NPs. Once NPs are formed, polyphenolic compounds are absorbed onto their surface, providing stability to the nanomaterials. The total flavonoid content was determined using a standard curve with quercetin (1.56–25.00 µg mL^−1^) and expressed as milligrams of quercetin equivalents (Q mL^−1^ of extract). The equation for the calibration curve was as follows:

36.329(±0.312)x − 0.0196 (±0.004), R^2^ = 0.9998, F = 13511.05, se = 0.479 (4)

The LOD for Q was determined to be 0.33 μg mL^−1^, calculated using the equation: LOD = 3 sd/S, where sd is the standard deviation of a blank and *S* is the slope of the calibration curve.

Quantification of total polyphenols was performed using a calibration curve with gallic acid (GA) at concentrations from 12.3 to 100 µg mL^−1^. The equation for the calibration curve was as follows:

7.591(±0.481)x + 0.170 (±0.028), R^2^ = 0.9920, F = 249.193; se = 0.259 (5)

The LOD for GA was determined to be 3.69 μg mL^−1^, calculated using the equation: LOD = 3 sd/S, where sd is the standard deviation of a blank and *S* is the slope of the calibration curve. The results were expressed as mg GA equivalents per ml of sample.

Total polyphenols and flavonoids, expressed as equivalents of GA and Q, respectively, are summarised in Table 2.

**Figure 3:**
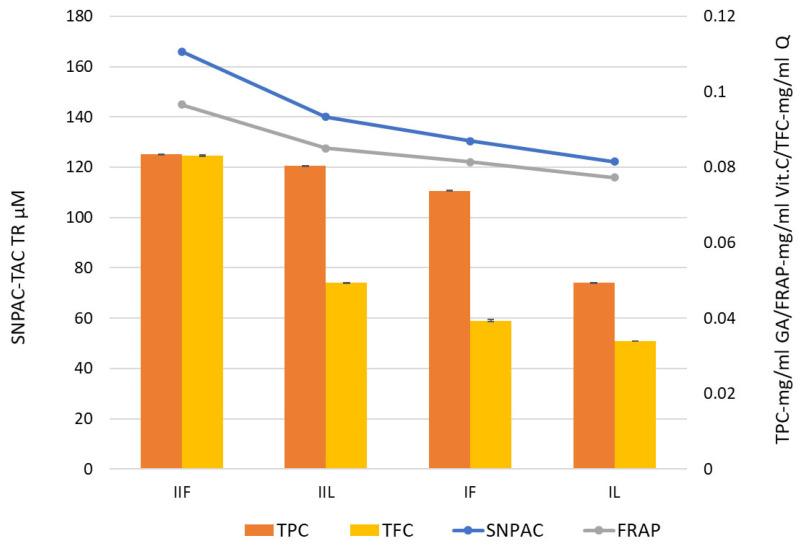
Total phenolic content (TPC) (mg GA mL^−1^) and total flavonoid content (TFC) (g Q mL^−1^) together with antioxidant activities measured by the use of FRAP (mg vit.C mL^−1^) and SNAP (TAC µM TR) methods for *Tanacum vulgare* L. extracts.

As regards the content of each group of compounds, both types of extracts (leaf and flower) showed a greater amount of total phenolic compounds than flavonoids. Figure 3 shows that the higher the content of phenolic compounds, the higher the antioxidant activity of the extract.

### 2.2. Tanacetum Vulgare-Ag-NPs Characteristic

#### 2.2.1. UV–vis Spectroscopy

A series of colloids of different colours was obtained using different types of extracts from both flowers and leaves of tansy (water, ethanol–water) and different volume ratios of 1 mM silver nitrate to extract (9:1, 1:1, and 1:9).

**Figure 4:**
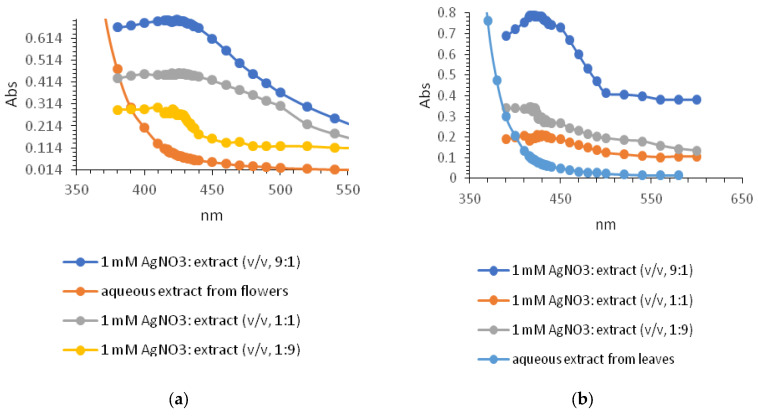
UV–visible absorption spectra of *Tanacetum vulgare* aqueous extracts from flowers (**a**) and leaves (**b**) together with *Tanacetum vulgare*-Ag-NPs synthesised by the use of different volume ratios of 1 mM AgNO_3_: extract (9:1, 1:1, and 1:9).

Monitoring of the reaction by UV–vis spectroscopy in the range fixed between 300 and 600 nm revealed a distinct, high-intensity absorption band around 420 nm, characteristic of Ag-NPs. It should be emphasised that the UV–vis spectrum of the extracts lacks the band responsible for surface plasmon resonance (SPR) (Figure 4a,b). It is clear that both the composition of the mixture and the types of extracts can directly influence the synthesis, growth, quantity, and quality of the NPs produced. The plasmon intensities of the bands varied according to the proportions of the components. The results showed that a volume ratio of 9:1 (*v*/*v*, 1 mM AgNO_3_/water extract) gave the highest absorption intensity, indicating a better reaction efficiency. For ethanol–water extracts, the band at 423 nm was not as pronounced, so they were excluded from further investigation. The formation of Ag-NPs was further confirmed by scanning electron microscopy (SEM), energy-dispersive X-ray spectroscopy, and Fourier transform infrared spectroscopy (FT-IR).

#### 2.2.2. Scanning Electron Microscopy (SEM) with Energy-Dispersive X-ray Spectroscopy (EDX)

**Figure 5:**
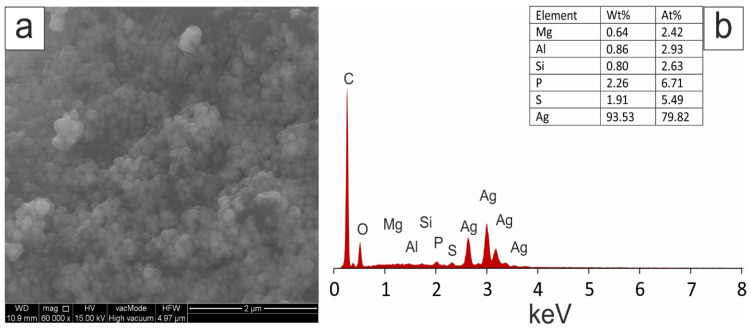
(**a**) SEM image with EDS analysis, and (**b**) analysis of Ag-NPs obtained using *Tanacetum vulgare* aqueous extracts from flowers.

**Figure 6:**
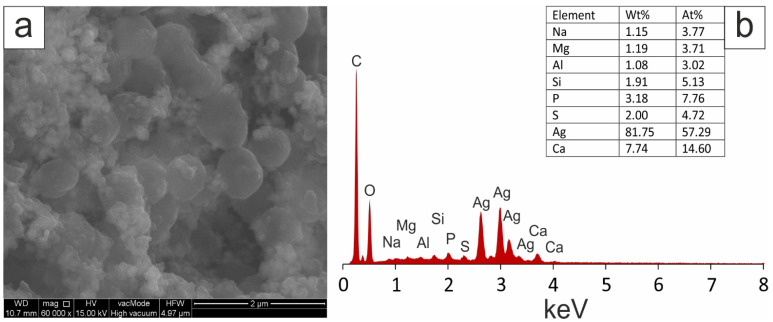
(**a**) SEM image with EDS, and (**b**) analysis of Ag-NPs obtained using *Tanacetum vulgare* aqueous extracts from leaves.

SEM images of separated and prepurified silver nanoparticles obtained by green synthesis from a mixture of 1 mM AgNO_3_ and aqueous extracts of tansy flowers and leaves in a ratio of 9:1 (*v*/*v*) showed mostly spherical nanoscale particles, as shown in Figure 5 and Figure 6. Elemental analysis was performed by EDX. A strong peak signal was observed at around 3 KeV, typical of metallic silver nanoparticles. The image shows agglomerates of fine grains. The images confirmed that the biosynthesised silver particles were nanoscale.

#### 2.2.3. Zeta Potential and Size of Ag-NPs

The ζ-potential is a measure of the stability of a colloidal suspension according to the Derjaguin–Landau–Verwey–Overbeek (DLVO) theory [123]. Its value depends on the surface charge of the particles and the type and concentration of ions present in the liquid dispersion medium. The more positively or negatively charged the particles are, the more they tend to repel each other and the more stable they are, resisting flocculation, agglomeration, or aggregation. In general, nanoparticles with a ζ potential greater than 30 mV or less than −30 mV are considered stable colloids [124,125]. At the same time, a potential range for stable dispersion of 20–30 mV is also suggested [126]. In practice, this value may vary depending on the system studied, the type of particles, and the experimental conditions. Some reports show a ζ-potential value of −22 mV as a criterion for the stability of silver nanoparticles [127]. Very low values ranging from −8 mV to −5 mV are found for NPs formed from chitosan (CS) and carboxymethylcellulose (CMC) [128], or high values when the surface of the NPs is additionally functionalised [129].

The negative ζ-potentials of the Ag-NPs obtained were −23.30 mV and −27.38 mV, respectively, for Ag-NPs synthesised using aqueous extracts of tansy flowers and leaves, suggesting that electrostatic repulsive forces are involved in the stabilisation of the aqueous dispersion. However, it should be noted that stability in nanoparticle dispersions can be enhanced not only by a mechanism based on electrostatic interactions but also by steric repulsion. In addition to the ζ-potential, other parameters such as particle size distribution, polydispersity, and particle morphology are important in assessing the stability of nanoparticles. The polydispersity index (the square of the standard deviation divided by the mean particle diameter, PDI = (σ/d)^2^) describes the width or spread of the particle size distribution. The PDI value can vary between 0 and 1 [130]. In this study, PDI values of 0.24 and 0.22 were obtained for Ag-NPs synthesised using aqueous extracts of huckleberry flowers and leaves, respectively, which is a rather low indicator of polydispersity. A PDI greater than 0.5 or approaching unity would indicate a high degree of polydispersity. Only a PDI value of less than 0.1 can be considered as a monodispersed particle size distribution.

**Figure 7:**
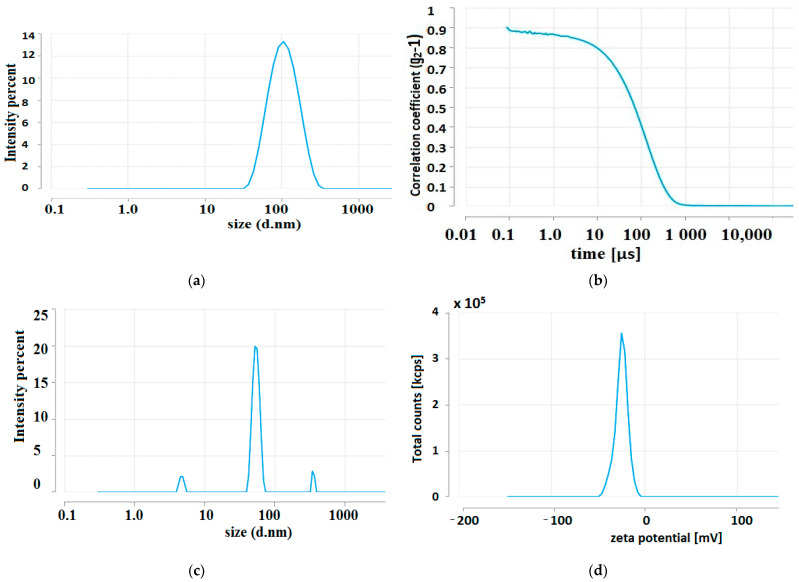
DLS analysis of the average size of Ag-NPs synthesised using 1 mM AgNO_3_ reduced with aqueous extracts of flowers of *Tanacetum vulgare* L. in a volume ratio of 9:1 (**a**); a plot of the intensity autocorrelation coefficients as a function of correlation time (**b**); the average size of Ag-NPs after 100-fold dilution of the colloidal mixture (**c**); and ζ-potential distribution (**d**).

The size distribution of Ag-NPs by intensity and the shape of the correlogram (Figure 7a,b) for the tansy flower extract indicated the presence of NP populations with regular, spherical shapes with an average hydrodynamic diameter of about 100 nm and more (Figure 7a). The zeta potential oscillated around −22.15 mV to −24.32 mV (Figure 7d), indicating sorption of charged extract components that stabilise the NPs and prevent aggregation. However, after 100-fold dilution, it was found that there are actually three populations of NPs of different sizes (Figure 7c, Table 3), the smallest of which, with dimensions of about 10 nm, has the highest concentration of 7.408 × 10^13^; the second peak concentration is 5.921 × 108, and the largest NPs have the lowest concentration of 2.92 × 10^4^ particles per mL.

**Figure 8:**
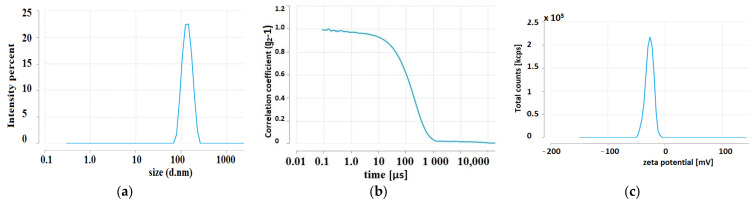
DLS analysis of the average size of Ag-NPs synthesised using aqueous extracts of leaves of *Tanacetum vulgare* L. (**a**); a plot of the intensity autocorrelation coefficients as a function of correlation time (**b**); and ζ-potential distribution (**c**).

NPs prepared from an aqueous extract of tansy leaves form a fairly homogeneous population of NPs with an average hydrodynamic diameter of about 147 nm (Figure 8a,b). The coefficient of variation of the Ag-NPs, which is the ratio of the SD (standard deviation) to the mean hydrodynamic diameter, was 0.58 (Figure 8c, Table 4).

#### 2.2.4. FT-IR Measurements

**Figure 9:**
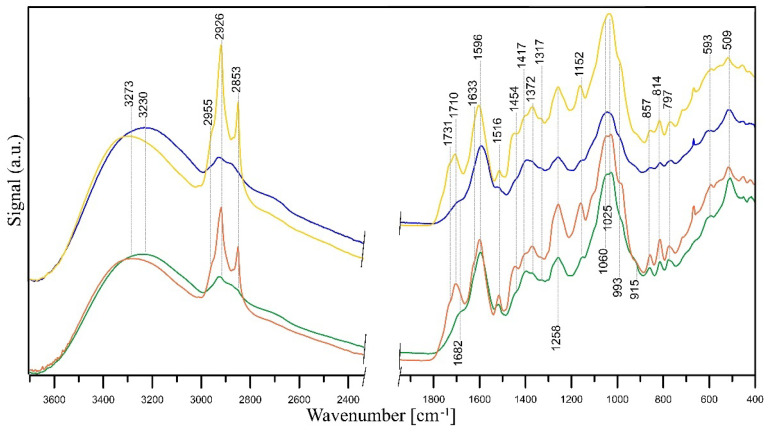
FT-IR/ATR spectra of the *Tanaceti flos* aqueous (green line) and hydroalcoholic (orange line) extracts and *Tanaceti folium* aqueous (blue line) and hydroalcoholic (yellow line) extracts.

**Figure 10:**
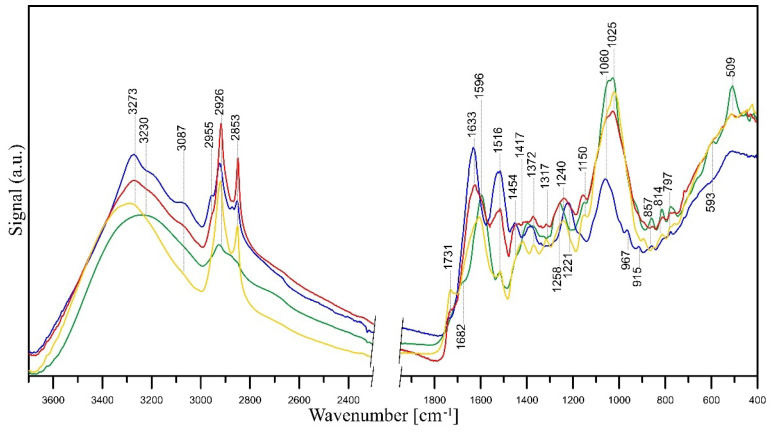
FT-IR/ATR spectra of the *Tanaceti flos* aqueous extract (green line) and related Ag-NPs obtained from a mixture of 1 mM AgNO_3_ and aqueous extracts of tansy flower in a ratio of 9:1 (*v*/*v*) (blue line), 1:1 (*v*/*v*) (red line), and 1:9 (*v*/*v*) (yellow line).

**Figure 11:**
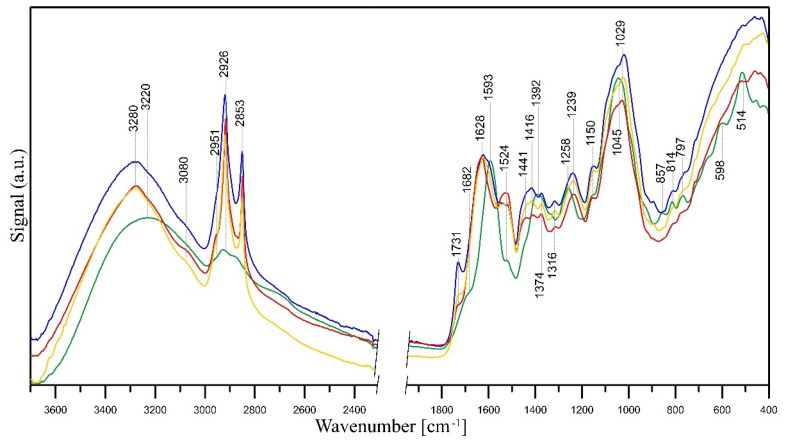
FT-IR/ATR spectra of studied samples: *Tanaceti folium* aqueous extract (green line) related Ag-NPs obtained from a mixture of 1 mM AgNO_3_ and aqueous extracts of tansy leaves in a ratio of 9:1 (*v*/*v*) (blue line), 1:1 (*v*/*v*) (red line), and 1:9 (*v*/*v*) (yellow line).

As mentioned, extracts made from flowers of tansy (*Tanaceti flos*) and tansy leaves (*Tanaceti folium*) contain essential oils of variable compositions (containing, i.a., beta-thujone, isomers, terpenes, camphene, and beta-pinene), polyphenols, amino acids, proteins, flavonoids (derivatives of quercetin and luteolin), oxygenated monoterpenes, monoterpene hydrocarbons, alcohols, carbohydrates, enzymes, and mineral compounds [87,131,132]. The presence of hydroxyl groups in those molecules is associated with the stabilisation and reduction of Ag^+^ ions to Ag^0^ [133]. Its further reduction leads to the formation of silver nuclei, resulting in the production of silver nanoparticles [134]. The FT-IR studies revealed that the carbonyl groups, i.e., from the amino acid residues and proteins, may possess significant potential to bind metal, suggesting that the proteins could put a stop to the aggregation that stabilises the medium [135]. FT-IR analysis can be therefore very useful in identifying biomolecules responsible for reducing Ag^+^ ions and stabilising synthesised Ag-NPs [132], and such analysis was performed. A comparison between the FT-IR spectra of *Tanaceti flos* and *Tanaceti folium* extracts and that of the synthesised silver nanoparticles can reveal the functional groups that are involved in the surface coating and effective stabilisation of the produced nanoparticles [136]. Figure 9 presents FT-IR/ATR spectra of *Tanaceti flos* and *Tanaceti folium* aqueous and hydroalcoholic extracts. Figure 10 and Figure 11 present the FT-IR/ATR spectra of *Tanaceti flos* and *Tanaceti folium* aqueous extracts and synthesised silver nanoparticles with different *v*/*v* ratios, respectively.

Analysing the IR spectra of the initial aqueous extracts from *Tanaceti flos* and *Tanaceti folium*, it can be concluded that their composition is similar, although the bands indicating the presence of amino acids (amide II band at ~1517 cm^−1^ and C–N stretching vibrations in amines at 1027 cm^−1^) have a higher intensity in the IR spectrum of *Tanaceti flos* extract, which indicates a higher content of these compounds. Bands at ~3280 cm^−1^ represent the stretching vibration of both the hydroxyl groups of carbohydrates and N–H in amino groups of proteins [137]. The presence of proteins provides dimensional stability and allows the nanometric structure to be kept stable in the aqueous medium without the need to use surfactants to avoid agglomeration of nanoparticles [138]. A wide band within 3600–3400 cm^−1^ may also be attributed to the nondissociatively adsorbed water molecules, the existence of which can be confirmed by the presence of a band at ~1630 cm^−1^ due to the deformation vibration of the water molecules. The weak band at ~3080 cm^−1^ is probably due to C–H vibration in vinyl =CH_2_ groups and/or N–H stretching vibration. Stretching vibrations of aliphatic C–H were observed within 2955–2853 cm^−1^ and at ~1416 cm^−1^ [137]. The latter band may be also assigned to C–OH deformation vibration with the involvement of the symmetric stretching vibration of O–C–O in carboxylate group.

The polyols and phenols may act as reducing agents, whereas some proteins and metabolites with functional groups such as amine, alcohol, ketone, aldehyde, and carboxylic may act as capping and stabilising agents. The bands of these groups are visible in the IR spectra of the extracts. The presence of ketones, aldehydes, quinines, and esters could be indicated by the peaks between 1700 and 1600 cm^−1^ and are assigned to the C=O vibration of carbonyl groups and/or C=C vibrations of aromatic structures. The absorption peaks at ~1595 cm^−1^ and ~1392 cm^−1^ in the extracts spectra point to COO– asymmetric and symmetric stretching, respectively [139]. The latter peak can also be assigned to C–OH stretching vibrations of the phenolic and/or alcoholic groups, while the peak at 1258 cm^−1^ is responsible for C–O stretching vibrations [140]. The CO stretching vibrations in quinine structures may also appear within this range [141]. The bands in the 1300–1000 cm^−1^ range were also described as substances with ether linkages, such as 1,8-cineole [139]. A broad band within 1060–1025 cm^−1^ is attributed to C–O–C stretching vibrations of aromatic ethers and polysaccharides. The peak around 1150 cm^−1^ may be attributed to the C–N stretching vibration of aromatic primary and secondary amines, and the bands within 900–600 cm^−1^ correspond to primary and secondary amines and amides (–NH_2_ wagging) and/or C–O and C–O–C symmetric stretching. The bands at ~598 cm^−1^ and ~510 cm^−1^ are C–CO–C and C–CO inplane deformation vibrations in ketone groups, respectively [137]. Analysing the spectra in Figure 9, it can be seen that the intensity of the bands of all functional groups in the spectra of hydroalcoholic extracts is higher than in the case of aqueous extracts. This means that the antioxidant activity is higher in the case of hydroalcoholic extracts compared to aqueous ones, which was confirmed using the SNAPC, FRAP, and DPPH methods.

The shift of some peaks and the appearance of new peaks in the spectra of Ag-NPs confirms the bioreduction of silver ions by specific functional groups of plant extract (Figure 10 and Figure 11). A shift of the bands of phenolic groups from ~1395 cm^−1^ to 1441 cm^−1^ and 1258 to ~1240 cm^−1^, a shift of the bands of ether groups from 1060 cm^−1^ to 1025 cm^−1^ (*Tanaceti flos* extract) and 1045 cm^−1^ to 1029 cm^−1^ (*Tanaceti folium*), and the disappearance of C–CO–C and C–CO inplane deformation vibrations in ketone groups (~598 cm^−1^ and ~510 cm^−1^) prove the reduction of Ag^+^ ions to Ag^0^ by means of these groups. Additionally, in the spectra of nanoparticles, the bands of COOH carboxyl groups (~3280 cm^−1^, 1731 cm^−1^) appear, which are the result of oxidation of phenolic groups. A slight shift or decrease in the intensity of the mentioned bands and an increase in the intensity of amide II band at ~1520 cm^−1^ and amine C–N stretching vibrations at 1027 cm^−1^ in the nanoparticles spectra indicate that proteins extracted from *Tanaceti folium* and *Tanaceti flos* coat the silver nanoparticles and stabilise them.

This stabilisation effect is best seen when the spectra of *Tanaceti folium* (Figure 11) are analysed. In turn, the changes in the spectra of nanoparticles obtained from *Tanaceti flos* are not so unambiguous, although the appearance of bands of COOH groups indicates the reduction of Ag^+^ ions (the most intense band of these groups is visible in the spectrum of sample with 1:9 (*v*/*v*) ratio (Figure 10, yellow line); the stabilising effect for this sample cannot be confirmed analysing the intensity of amide groups). On the other hand, the intensity of the band of amine groups (1025 cm^−1^) is rather high. In the spectrum of the sample with 9:1 (*v*/*v*) ratio (Figure 10, blue line), the high intensity of the band at 1516 cm^−1^ indicates the coverage of the nanoparticles with amide groups and their stabilisation (the amine groups band at 1025 cm^−1^ has very low intensity), while the low intensity of the bands of COOH groups may indicate a lower degree of silver ion reduction [142].

### 2.3. Kinetics of Ag+ Ion Release from Ag-NPs in Aqueous Suspensions

**Figure 12:**
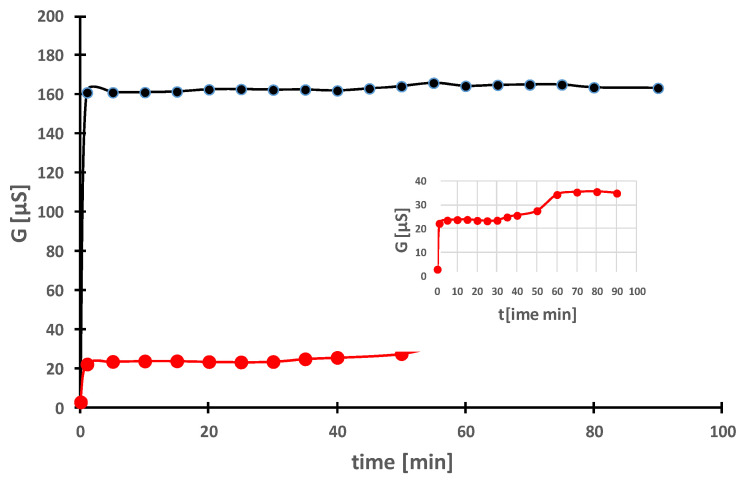
Changes in conductivity (µS) of the aqueous suspension of Ag-NPs at a mass concentration of 400 mg L^−1^ versus time in (min). Ag-NPs were prepared using an aqueous extract of *Tanaceti folium* and 1 mM AgNO_3_ (1:9, *v*/*v*). Ag-NPs were separated after centrifugation and dispersed in deionised water without washing (black line) or washed and redispersed in deionised water (red line, inset).

Despite the fact that the number of studies on the bioactivity of Ag-NPs has increased steadily, especially in the last 10–15 years, research on the mechanism of action is a matter of recent years [143,144,145,146]. Ag-NPs are known to be cytotoxic [147], partly due to the release of Ag^+^ ions [145,146,148,149,150]. Ag^+^ ions can be released into the dispersion medium but also into cells. This process depends on many factors, such as temperature, pH, oxygenation, and the presence of other ions [149,150,151,152,153,154,155]. The size of Ag-NPs [144,146,151] and surface functionalisation [156] also play a significant role in influencing the kinetics of Ag+ ion release. Therefore, to confirm the involvement of ions in the mechanism of action of the obtained NPs, the release of Ag^+^ ions from Ag-NPs in aqueous solutions was monitored by conductivity measurement. Ag^+^ ions have a rather high value of the limiting molar conductivity Λ_0_ = 61.9 S cm^2^mol^−1^, thanks to which their release affects the measurement. Figure 12 shows the change in conductivity of aqueous dispersions containing 400 mg L^−1^ Ag-NPs as a function of time for two types of Ag-NPs prepared by reducing the AgNO_3_ starting solution with aqueous extracts of tansy leaves without and with washing (the dispersed Ag-NPs are removed by centrifugation and redispersed in fresh deionised water) and without any stirring.

The relationship between conductivity (G) and time reached its asymptotic value immediately (less than 1 min) for the dispersion containing unwashed Ag-NPs. This almost immediate dissolution within minutes contrasts with the dissolution of washed Ag-NPs, where the conductivity increased, reaching the first plateau after 20 min and the second plateau after one hour. After one hour, the conductivity had not changed at all and remained at the constant level of ∼35 µS. The first equilibrium probably reflects the release of ions from the surface coatings, while the second is an effect of the dissolved nanosilver coming from the metal–oxygen reaction. The release of ions from washed nanosilver is almost 4.5 times lower compared to unwashed Ag-NPs.

Similar effects have been observed by other authors, e.g., in the case of modification of Ag-NPs with polyvinylpyrrolidone [149] or citrate [149,150], due to the presence of surface coatings, which on the one hand limit the release and on the other hand prevent agglomeration.

In our study, the slow release of ions (second plateau) may be related to surface functionalisation or the presence of sulphur (Figure 5 and Figure 6), which can form insoluble sulphides with silver on the nanosilver surface, leading to slow release. Some authors associate this reaction with the inhibition of antibacterial activity [155,156,157,158].

### 2.4. Antimicrobial Activity

Antimicrobial activity of the prepared Ag-NPs against selected bacteria, fungi, and yeasts was tested in comparison to aqueous extracts of tansy flower and leaves. Ag-NPs were prepared by green synthesis from a mixture of 1 mM AgNO_3_ and aqueous extracts in a ratio of 9:1 (*v*/*v*). The above conditions ensured the most stable and the smallest NPs.

The results of the antibacterial and antifungal activities are presented in Table 5. Aqueous extracts of the *Tanaceti flos* and *Tanaceti folium* were found with weak bioactivity (MIC > 1000 mg L^−1^) against tested reference strains. Noticeably, the tested Ag-NPs obtained in green synthesis using either the *Tanaceti flos* or *Tanaceti folium* showed good bioactivity against both Gram-negative bacteria (*E. coli, S. Typhimurium, K. pneumoniae,* and *P. mirabilis*) and Gram-positive bacteria (*S. aureus*, *Methicillin-resistant Staphylococcus aureus (MRSA)*, *B. cereus*, and *E. faecalis*) with a minimal inhibition concentration range from 31.3 to 62.5 mg L^−1^. The MIC values for Gram-positive reference bacteria indicated strong (MIC 15.6 mg L^−1^) anti-*S. epidermidis* and very strong anti-micrococcal (*M. luteus*) activity (MIC 7.8 mg L^−1^). *Tanaceti flos* Ag-NPs as well as *Tanaceti folium* Ag-NPs also had strong activity against *P. aeruginosa* (MIC 15.6 mg L^−1^). Both Ag-NPs obtained in green synthesis were mostly bactericidal against tested strains, showing an MBC/MIC index ≤ 4. Antifungal bioactivity (*C. albicans*, *C. glabrata*, and *C. parapsilosis*) was not observed for aqueous extracts (MIC > 8000 mg L^−1^); however, *Tanaceti flos* Ag-NPs as well as *Tanaceti folium* Ag-NPs were strongly and very strongly active, respectively, against the *Candida* spp. tested.

### 2.5. Anticancer Activity

#### 2.5.1. HeLa Cancer Cells

The cytotoxicity study showed that both extracts are significantly less toxic than Ag-NPs obtained by green synthesis to both normal Vero and HeLa cancer cells (Table 6). The CC50 of the flos and folium extracts for the Vero and HeLa lines fell in the ranges of 596.6–861.3 µg mL^−1^ and 681.0–753.1 µg mL^−1^, respectively. In turn, the CC50 of respective Ag-NPs for the Vero line was 14.1–22.1 µg mL^−1^, and for the HeLa line, 57.9 − 67.3 ± 0.2 µg mL^−1^. Evaluation of the cytotoxic activity showed that the investigated extracts as well as Ag-NPs obtained using green synthesis do not have anticancer potential. The selectivity index (SI) was below 1 for most samples; only *Tanaceti folium* extract was slightly above 1 (SI = 1.1). The SI value above 1.00 means a higher selectivity of the extract for cancer cells than for normal cells.

#### 2.5.2. Melanoma Cells

**Figure 13:**
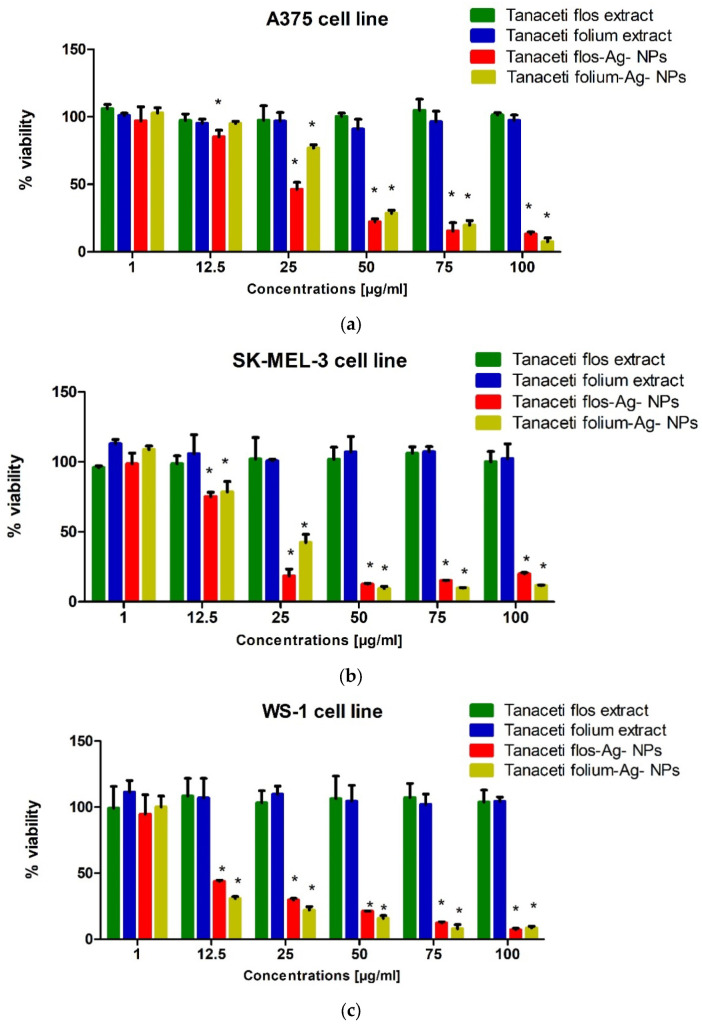
Evaluation of the effect of the tested extracts on the viability of A375 (**a**), SK-MEL-3 (**b**), and WS-1 (**c**) cells after 24 h incubation with *Tanaceti flos* extract, *Tanaceti folium* extract, *Tanaceti flos* Ag-NPs, and *Tanaceti folium* Ag-NPs. Results are presented as mean % cell viability ± SD relative to control (cells not treated with any extract). Significant values (*) compared to control at *p* < 0.05 (one-way ANOVA with Tukey’s post hoc test).

In order to determine changes in the viability of human melanoma cells (A375, SK-MEL-3) and human fibroblasts (WS-1) after the application of specific concentrations of the tested extracts, the MTT test was performed. Based on the results, it can be concluded that *Tanaceti flos* extract and *Tanaceti folium* extract at concentrations of 1–100 µg mL^−1^ did not show statistically significant changes in the viability of the cell lines used in the experiment (Figure 12). *Tanaceti flos* Ag-NPs caused statistically significant changes in the viability of melanoma cell lines already at a concentration of 12.5 µg mL^−1^ (Figure 13), while *Tanaceti folium* Ag-NPs statistically significantly reduced the viability of A375 cells at a concentration of 25 µg mL^−1^ and higher (Figure 13a), and SK-MEL- 3 at a concentration of 12.5 µg mL^−1^ and higher (Figure 13b). The decrease in cell viability increased with the increase in the concentration of extracts. The SK-MEL-3 cell line derived from melanoma metastases was more sensitive to *Tanaceti folium* Ag-NPs and *Tanaceti flos* Ag-NPs. *Tanaceti folium* Ag-NPs and *Tanaceti flos* Ag-NPs also strongly affected the decrease in viability of WS-1 cells; a statistically significant decrease in viability was observed at a concentration of 12.5 µg mL^−1^ and higher (Figure 13c). Approximate IC50 values for the tested extracts are presented in Table 7.

## 3. Discussion

Many reports have shown that Ag-NPs synthesised from plant extracts have diverse antibacterial [142,159] and anticancer activities [160,161,162,163]. The mechanism of action of Ag-NPs is not fully understood, although it is known to depend on, among other things, their size, shape, and exposure time [164,165]. Published interpretations consider both the contribution of cations released by Ag-NPs and that of zero-valent metallic nanoparticles. Of particular relevance to modern medicine is research into the potential of nanoparticles to combat the most dangerous pathogenic bacteria, such as *E. faecalis*, *S. aureus*, *Staphylococcus epidermidis*, *E. coli*, *Klebsiella pneumoniae*, and *P. aeruginosa* [166], which are capable of forming biofilms that are resistant to antibiotic therapy [167]. Reports confirm the efficacy of Ag-NPs to inhibit biofilms of *P. aeruginosa* and *S. epidermidis* [168], *Klebsiella pneumoniae* [169], methicillin-resistant *Staphylococcus aureus* [170] and *Mycobacterium tuberculosis* [171], *E. coli*, *S. aureus*, and *Candida albicans* [172].

In the present work, Ag-NPs were synthesised using aqueous and hydroalcoholic extracts of tansy as reducing agents, stabilising agents, and precursor molecules for Ag-NP formation. Previously, it was claimed that the nanoparticles developed from the different plants of the *Asteraceae* family are highly stable, nontoxic, and environmentally friendly [173]. So far, the green synthesis of silver and gold nanoparticles mediated by tansy extracts has been described by Dubey et al. [174]. The experiments performed showed that the hydroalcoholic extracts of tansy flowers had the highest reducing power. This was due to the higher content of polyphenols, mainly flavonoids, which can act as reducing agents in green synthesis.

The FT-IR/ATR spectra confirmed that the bands of the hydroalcoholic extracts were more intense than those of the aqueous extracts. This was in agreement with the SNAPC, FRAP, and DPPH results. The total phenolic content of the extracts, determined by the Folin–Ciocalteu method, increased in the following order: water–alcohol extract of tansy flowers > water extract of tansy flowers > water–alcohol extract of tansy leaves > water extract of tansy leaves. This order is consistent with the results reported by other authors [93]. However, the total phenolic content obtained in our study, which varied from 49.43 to 83.46 µg GA mL^−1^ extract, is an order of magnitude lower compared to the data reported by Mot et al. [93], probably due to the different habitats of *Tanacetum vulgare* L. as well as the lower temperature and shorter extraction time. Tansy extracts were used for the phytogenic synthesis of NPs with unique shape, size, and monodispersity in the solution phase. In this study, the most favourable physicochemical parameters, i.e., size, ζ-potential, and shape, were obtained for NPs synthesised with aqueous tansy extracts, and therefore, they were used for further bioactivity tests. The bioreduction of silver ions by specific components of the plant extracts was confirmed by comparing the spectra of Ag-NPs with the corresponding FT-IR/ATR spectra of the extracts.

The IR spectra revealed the involvement of hydroxyl groups of carbohydrates and amino groups of proteins in providing stability to the nanometric structure. On the other hand, phenolic, ether, and ketone groups seem to be responsible for the bioreduction of Ag^+^ ions to Ag^0^. Moreover, a lower content of extract implies a rather low reducing power, as can be seen by comparing the spectrum of the mixture containing the 9:1 (*v*/*v*) ratio of 1 mM AgNO_3_: plant extract with the mixture with the reversed 1:9 (*v*/*v*) ratio. However, it should be stressed that despite the rather low reducing power, the NPs are stable due to the coverage of the nanoparticles with amide groups.

Similar to other works, *Tanaceti flos* Ag-NPs and *Tanaceti folium* Ag-NPs showed good bioactivity against *Candida* spp. And against both Gram-negative bacteria (*E. coli*, *S. Typhimurium*, *K. pneumoniae*, and *P. mirabilis*) and Gram-positive bacteria (*S. aureus*, *B. cereus*, and *E. faecalis*) with minimum inhibitory concentrations ranging from 31.3 to 62.5 mg L^−1^. In contrast, aqueous extracts of *Tanaceti flos* and *Tanaceti folium* were found to have low bioactivity. The results of another study show in vitro antimicrobial activity of biologically active compounds in *Tanacetum vulgare* extracts against *B. subtilis*, *P. aeruginosa* and *E. coli*, *C. albicans*, *B. subtilis*, *P. aeruginosa*, and *E. coli* [175]. The extract of *Tanacetum vulgare* shows antimicrobial properties and has the ability to inhibit biofilm synthesis, as shown by Devrnja et al. [176]. Another study [112] also reported the antibacterial activities of *Tanacetum vulgare* hydroethanol extracts. This is inconsistent with the lack of antimicrobial activity of the aqueous extracts obtained in our study. The difference can be explained by the different habitats of *Tanacetum vulgare* and mainly by the method of extract preparation, which in study [175] was prepared by the Soxhlet method using methanol in an acid medium for 15 cycles over a period of up to 8 h. In addition, the authors looked separately at the antimicrobial activity of individual biologically active substances extracted from *Tanacetum vulgare*, rather than the extract as a whole.

Two mechanisms of antibacterial activity of Ag-NPs have been proposed. The first mechanism involves the effect of direct contact of NPs with microorganisms and the second, killing by released ions [142]. The anchoring of Ag-NPs in the bacterial cell wall, which leads to its damage [177,178], can be facilitated by an appropriate surface charge. Thus, the antibacterial activity of Ag-NPs is the result of electrostatic attraction between Ag-NPs and the negatively charged cell membrane of microorganisms due to the presence of carboxyl, phosphate, and amino groups [179,180]; in our case, the negative charge of Ag-NPs does not seem to be favourable from the point of view of the contact-killing mechanism. In the context of the studies carried out on the stability of Ag-NPs in aqueous dispersion systems, the effect of silver ions released either from the surface of Ag-NPs or from nanosilver as a product of oxidation cannot be excluded. It should be emphasised that the contribution of ions to the mechanism of both anticancer and antimicrobial activity will be greater if Ag-NPs are not washed from excess ions with deionised water. The synthesised Ag-NPs are active against both Gram-negative and Gram-positive bacteria without any specific difference. The strongest activity of the synthesised Ag-NPs was observed against yeast. Ag-NPs can also cross the membrane and enter bacteria. Smaller nanoparticles are preferred because they pass through bacterial cells more easily than larger ones [178]. There is evidence that silver ions (Ag^+^) released from Ag-NPs are also responsible for the antibacterial activity [181,182,183]. Again, smaller NPs with the largest surface area are preferred, ensuring the highest concentration of Ag+ released [184]. The mechanism of antibacterial action of silver ions is complex and involves, among other things, protein deactivation due to the binding of silver ions to sulfhydryl groups; initiation of ROS production [185]; and the breaking of the H-bond between base pairs of antiparallel DNA strands [186,187,188]. The synergistic effect of Ag-NPs with antibiotics appears to be particularly beneficial for infections caused by multiresistant bacterial strains.

The cytotoxicity study showed that both extracts and green-synthesised Ag-NPs are toxic to both normal Vero and HeLa cancer cells. However, it should be emphasised that the extracts are significantly less toxic than the Ag-NPs. This is also noted by other authors reporting cytotoxicity and genotoxicity of small-size nanoparticles, especially at high concentrations [189]. Green-synthesised Ag-NPs were also cytotoxic in the case of the melanoma cell lines (A375, SK-MEL-3). However, even greater cytotoxicity was observed in the reference human fibroblasts (WS-1). Thus, although the cytotoxicity observed was cell-type dependent, it was strong in all lines tested. Considering that the cytotoxicity could be a direct consequence of the oxidative stress induced by Ag-NPs and the release of Ag ions, the observed differences suggest the strong influence of the surface modification as a result of the phytogenic synthesis. Certainly, the surface modification of nanoparticles achieved by green synthesis using *Tanacetum vulgare* extracts affected the interaction of Ag-NPs with cells. It is known that Ag-NPs without surface modification agglomerate and do not migrate to the nucleus and mitochondria. Surface coats of macromolecules such as polysaccharides and proteins allow such translocation [190,191].

## 4. Materials and Methods

### 4.1. Materials

All the chemicals were of analytical grade. 1,1-Diphenyl-2-picrylhydrazyl free radical (DPPH), ascorbic acid (vitamin C), chlorogenic acid (CGA), Trolox (TR), and quercetin (Q) were purchased from Sigma-Aldrich (St. Louis, MO, USA). Silver nitrate (AgNO_3_) was purchased from Sigma-Aldrich Inc., St. Louis, MO, USA. Ethanol was purchased from E.Merck (Darmstadt, Germany). All aqueous solutions were prepared using water purified through an ULTRAPURE Millipore Direct-Q 3UV-R (Merck, Darmstadt, Germany) with a resistivity of 18.2 MΩ cm.

### 4.2. Collection of Plant Material and Sample Preparation

The aerial parts of *Tanacetum vulgare* L. plants were collected in southeastern Poland in August 2022. The plants were dried in the dark at room temperature. For extraction, 5 g of dried powder of flowers (F) or leaves (L) were suspended in 100 mL of deionised water (extract IF, extract IL) or 80% (*v*/*v*) ethanol (extract IIF, extract IIL) in a 250 mL Erlenmeyer flask and sonicated for 60 min in an ultrasonic bath (ultrasound power 1200 W, frequency 35 kHz) using a Bandelin Sonorex RK 103 H (Bandelin Electronics, Berlin, Germany) at 80 °C. After cooling, the extracts were centrifuged at 11,000 rpm for 15 min to precipitate traces of solids from the extract. The supernatants were collected, filtered through Whatman No. 1 filter paper, and stored in the refrigerator at 4 °C for further study.

### 4.3. Estimation of the Total Phenolic (TPC) and the Total Flavonoid Content (TFC)

Total flavonoid content (TFC) was determined spectrophotometrically using a Genesys 20 spectrophotometer (The ThermoSpectronic, Waltham, MA, USA) according to method described, among others, by the Lamaison and Carnat research group [65,192]. Briefly, 1.0 mL of 2% aluminium chloride (AlCl_3_) in water was mixed with 25 µL of extract and 975 µL of water. Absorbance readings at 415 nm were taken after 15 min against a blank (water). The TPC content was determined using a standard curve with quercetin (1.56–25.00 µg mL^−1^). The mean of three readings was used and expressed as milligrams of quercetin equivalents (Q mL^−1^ extract).

Total phenolic content (TPC) was determined by the Folin–Ciocalteau method [118,193] with slight modifications proposed by de Camargo et al. [194]. Briefly, 50 µL of extracts, 450 µL of deionised water, and 500 µL of diluted Folin–Ciocalteu phenol reagent from Sigma-Aldrich (St. Louis, MO, USA) (1 mL + 30 mL water) were added to flasks and mixed thoroughly. Then, 1.0 mL of 0.35 M NaOH solution was added, and the mixture was kept in the dark for 3 min at room temperature (23–25 °C). The absorbance was then read at 760 nm. Quantification of TPC was performed using a calibration curve with gallic acid (GA) (12.3–100 µg mL^−1^).

### 4.4. Antioxidant Activity Assays

#### 4.4.1. The Silver NanoParticle Antioxidant Capacity (SNPAC)

SNPAC measurements were performed according to the procedure described by Özyürek et al. [117] and tested in our previous investigation [195]. Briefly, the initial solution of small, spherical, silver nanoparticles (Ag-NPs) was prepared by adding 5 mL of freshly prepared 1% aqueous tripotassium citrate solution drop by drop to 50 mL of 1 mM silver nitrate heated to 90 °C in the dark with constant stirring using a magnetic stirrer until a pale yellow colour was obtained. The resulting Ag-NP solution was stored in the dark. Note that comparative measurements should be made on the same day using the same batch of Ag-NP starting solution, as its absorbance decreases by approximately 6.7% after 1 day of storage. The test samples were prepared by mixing 2 mL Ag-NPs + 10 µL standard or tested extract +7.99 mL water. After 30 min storage at 25 °C in the dark, the mixtures turned dark brown. The increase in absorbance was measured spectrophotometrically at 423 nm.

#### 4.4.2. Ferric Reducing Antioxidant Power (FRAP) Assay

The FRAP activity of the extracts was determined by a slight modification of the method of Cañadas et al. [196] and Oyaizu [197]. The FRAP solution was prepared using the following reagents: 300 mM sodium acetate trihydrate buffer pH 3.6; TPTZ (2,4,6-tri-2-pyridinyl-1,3,5-triazine) 10 mM in 40 mM HCl; and 20 mM FeCl_3_ hexahydrate. The working FRAP reagent was mixed in a ratio of 10:1:1 before the assays were performed. The standard solution was ascorbic acid dissolved in water. The different ascorbic acid dilutions were prepared as follows: 0.00625; 0.0125; 0.025; 0.05; and 0.1 mg/mL. For the tests, 1450 µL of FRAP reagent and 50 µL of standard were added and the samples were mixed for 15 s and measured at an absorbance of 593 nm. For the blank, the same procedure was followed using 1450 µL FRAP reagent and 50 µL distilled water.

#### 4.4.3. The 2,2-Diphenyl-1-Picrylhydrazyl (DPPH) Radical Scavenging Assay

This assay measures the disappearance of the purple colour of DPPH radicals in the presence of antioxidants. Changes in the initial absorbance at 517 nm of the radicals were obtained by adjusting the volume of the extracts. The DPPH assay used in this experiment is based on the protocol described by Hatano et al. [198] with minor modifications. Briefly, 1.5 mL of 1 mM DPPH ethanolic solution was well mixed with 20 µL of the twice-diluted extracts and then incubated in the dark for 30 min. Absorbance at 517 nm was measured in triplicate using a UV–vis spectrophotometer. The results were expressed as the IC50 value at which the sample reduces the absorbance intensity of DPPH free radicals by 50%. The IC50 values were converted to the concentration of chlorogenic acid used as the reference antioxidant (mg mL^−1^ extract).

### 4.5. Synthesis of Ag-NPs by Plant Extracts

The synthesis of Ag-NPs was carried out at 90 °C in the dark. The plant extracts were added dropwise to the appropriate amounts of AgNO_3_ aqueous solution (1 mM) in the ratio of 1:9, 1:1, or 9:1 with constant stirring at 800 rpm using a magnetic stirrer. The reaction mixture was then incubated in the dark at room temperature, and the progress of the synthesis was monitored periodically using a Thermo Scientific™ GENESYS™ 20 Visible Spectrophotometer in the wavelength range between 300 and 600 nm with a resolution of 1 nm. The appropriate dilutions of the extracts were used as blanks. The gradual colour change from colourless to yellow and finally to reddish-brown was observed after 30 min to 1 h. After 24 h of incubation, the obtained suspension was centrifuged at 12,000 rpm for 30 min (14,000 rpm/10 min) in a high-speed centrifuge (MPW-223e, MPW MED. INSTRUMENTS, Warsaw, Poland) after which the liquid supernatant was discarded and the solid pellets were washed with deionised water to remove silver ions and extract residues, frozen at −25 °C, and lyophilised. The powders were then stored in airtight glass vials at 4 °C until further analysis.

### 4.6. Characterisation of Ag-NPs

Ag-NPs were characterised by various analytical techniques including UV–vis spectroscopy using a Genesys 20 (The ThermoSpectronic, Waltham, MA, USA) spectrophotometer, and the size distribution of Ag-NPs was obtained using Zetasizer Ultra Red ZSU 3305 (Malvern Panalytical Ltd., Malvern, GB, USA), scanning electron microscopy (SEM), energy-dispersive spectrometry (EDS) analysis, and Fourier transform infrared (FT-IR) spectroscopy.

#### 4.6.1. SEM and EDS

The morphological forms and chemical composition of Ag-NPs were characterised by scanning electron microscopy (SEM) using a Quanta 250 FEG scanning electron microscope from FEI (Almelo, The Netherlands) equipped with energy-dispersive spectrometry (EDS). Liquid samples were dried prior to SEM analysis. Residues were dissolved in ethanol, and the solvent was evaporated in a vacuum dryer at 70 °C. The samples were then glued to carbon tape on an aluminium holder and sputtered with graphite.

#### 4.6.2. FT-IR

Fourier transform infrared attenuated total reflectance (FT-IR/ATR) spectra were recorded in the 4000–400 cm^−1^ range, resolution 4 cm^−1^, at room temperature, using a Nicolet 6700 spectrometer and a Meridian Diamond ATR accessory (Harrick). Dried samples were directly applied onto the diamond crystal, and close contact was made with the surface by a pressure tower. Interferograms of 512 scans were average for each spectrum. Dry potassium bromide (48 h, 105 °C) was used as a reference material to collect ATR spectra. All ATR spectra were corrected for water vapour and carbon dioxide, and ATR correction was applied. No smoothing functions were used. All spectral measurements were performed at least in triplicate.

#### 4.6.3. DLS

Dynamic light scattering (DLS) was used to estimate the average particle size and zeta potential of NPs. Measurements were performed using the Zetasizer Ultra DLS system (Malvern Panalytical, Malvern, UK).

#### 4.6.4. Conductivity Measurements

The kinetics of Ag+ ion release from Ag-NPs in aqueous suspensions was monitored using a multifunction meter CX-401 (Elmetron, Zabrze, Poland) with a Euro sensor EPS-2ZA (POCH, Gliwice, Poland). The unwashed Ag-NPs were obtained directly from the synthesis mixture (Tanaceti folium aqueous extract and 1 mM AgNO_3_), which was centrifuged, after which the liquid supernatant was discarded and the solid pellets were redispersed in deionised water. The washed Ag-NPs were obtained by centrifugation of their aqueous suspension of the solid pellets and, after removal of the supernatant, they were immediately redispersed in fresh deionised water without stirring. The conductivity was measured at ambient conditions for 1.5 h.

### 4.7. Antimicrobial Activity Assay

All compounds dissolved in deionised water were screened for antibacterial and antifungal activities by 2-fold microdilution broth method. Minimal inhibitory concentration (MIC) of tested compounds were evaluated for the panel of reference Gram-positive bacteria: *Micrococcus luteus* ATCC 10240, *Bacillus cereus* ATCC 10876, *Staphylococcus aureus* ATCC 25923, *S. aureus* ATCC BAA-1707, and *Staphylococcus epidermidis* ATCC 12228; Gram-negative bacteria: *Escherichia coli* ATCC 25922, *Proteus mirabilis* ATCC 12453, *Klebsiella pneumoniae* ATCC 13883, *Salmonella Typhimurium* ATCC 14028, and *Pseudomonas aeruginosa* ATCC 9027; and yeasts: *Candida albicans* ATCC 102231, *Candida parapsilosis* ATCC 22019, and *Candida glabrata* ATCC 90030. The procedure for conducting antimicrobial activity testing has been described in detail before [199].

### 4.8. Cytotoxicity Assay

#### 4.8.1. Cells

The Vero cell culture (ATCC CCL-81) and HeLa (ATCC CCL-2) were used in the experiment. The cells were cultivated in DMEM (Dulbecco’s Modified Eagle Medium, Corning, Tewskbury, MA, USA). For cell passaging, the culture media was supplemented with 10% foetal bovine serum—FBS (Capricorn)—whereas the media used for experiments contained 2% serum only. All media were supplemented with penicillin–streptomycin solution (100×) from Corning (Tewksbury, MA, USA). The cell culture was incubated at 37 °C in the 5% CO2 atmosphere. The cell lines used in the experiments were obtained from American Type Culture Collection (ATCC; Manassas, VA, USA). The human melanoma cell line A375 was cultured in high-glucose DMEM supplemented with 10% foetal bovine serum (FBS). The human melanoma cell line SK-MEL-3 was cultured in McCoy’s 5A medium supplemented with 15% FBS. The human fibroblast cell line WS-1 was cultured in EMEM medium supplemented with 10% FBS. The antibiotics penicillin (100 U/mL) and streptomycin (100 ug/mL) were also added to each culture medium. Cell lines were maintained under standard conditions (5% CO_2_, 37 °C). All reagents used in culture were purchased from Sigma Aldrich (St. Louis, MO, USA).

#### 4.8.2. Cytotoxicity Assay on HeLa Cells

Cytotoxicity of the tested compounds was estimated with the use of the MTT method [200]. Extracts were dissolved and further diluted with a complete test medium. An amount of 100 µL of the cell culture prepared was seeded into 96-well plastic plates (Becton Dickinson and Company, Franklin Lakes, NJ, USA) at a cell density of 1.5 × 10^4^ (Vero) and 2.5 × 10^4^ (HeLa) cells per well. After 24 h incubation at 37 °C, the media were removed and the cells were treated with a solution of the examined extracts diluted in the media with 2% serum. The cells were submitted to a series of compound concentrations, from 2000 µg/mL to 0.97 µg mL^−1^. Two-fold serial dilutions of compounds were added to the cells in triplicate. The cell cultures were incubated for 24 h at 37 °C in the 5% CO_2_ atmosphere. After 24 h incubation with extracts cell cultures were supplemented with 10 µL per well of 5 mg mL^−1^ MTT (Sigma-Aldrich, Saint Louis, MO, USA) stock in PBS (Corning) 10 µL per well, and the incubation was continued for 4 h at 37 °C. Then, 100 µL of aqueous solution containing 50% dimethylformamide (POCH, Gliwice, Poland) and 20% SDS (PanReac AppliChem, ITW Company, Darmstadt. Germany) was added to solubilise the insoluble formasane precipitates produced by MTT. After the all-night incubation, the absorbance was measured by the Synergy H1 Multi-Mode Microplate Reader (BioTek Instruments, Winooski, VT, USA) at two wavelengths—540 and 620 nm. On the basis of the test results, the cytotoxic concentration (CC50), which is the amount of tested substance that is required to reduce the number of viable cells by 50% compared to the control culture, was determined and was calculated using Gen 5 3.09.07 software (BioTek Instruments, Winooski, VT, USA). Cell viability (%) was calculated as (A540/620 of the treated/A540/620 of the control) × 100.

#### 4.8.3. Cytotoxicity Assay on Melanoma Cells

The viability of melanoma cells and fibroblasts was assessed using the MTT test (Sigma Aldrich, St. Louis, MO, USA). The MTT assay involves the reduction by metabolically active cells of the tetrazole salt (3-(4,5-dimethylthiazol-2-yl)-2,5-diphenyltetrazolium bromide, MTT) to insoluble formazan. In the first stage of the experiment, the cells were plated in 96-well culture plates (Nunc, Roskilde, Denmark). The density of the SK-MEL-3 cell suspension was 6 × 10^4^ cells/mL, the A375 cell line was 5 × 10^4^ cells/mL, and the WS-1 cell line was 1 × 10^5^ cells/mL. In the second stage of the experiment, after 24 h incubation, the culture medium was removed and the cells were exposed to serial dilutions of the test extracts (1, 12.5, 25, 50, 75, and 100 µg/mL) or fresh cell culture medium (control). The next step was a 24 h incubation of the cells with the test compounds under standard conditions. The next day, MTT solution (5 mg/mL) was added to the cells and incubated for 3 h. In the next step, 10% sodium dodecyl sulfate (SDS) solution was added and incubated overnight. Absorbance (wavelength 570 nm) was measured after 24 h using a microplate reader (Epoch, BioTek Instruments, Inc., Winooski, VT, USA) with Gen5 software (version 2.01, BioTek Instruments, Inc., Winooski, VT, USA). Stock of test extracts (100 mg/mL) was made by dissolving compounds in dimethyl sulfoxide (DMSO) (Sigma Aldrich, St. Louis, MO, USA). The resulting suspension in the appropriate proportion was added to the culture medium in the second stage of the experiment to obtain the tested concentrations.

### 4.9. Statistical Analysis

The GraphPad v.5.01 program (GraphPad Software, Inc., La Jolla, CA, USA) was used for the statistical analysis of the results. The data included in the charts are presented as means ± standard deviations (±SD) and were subjected to statistical analyses using the one-way ANOVA with Tukey’s post hoc test. IC50 values were calculated using an online IC50 calculator: https://www.aatbio.com/tools/ic50-calculator, accessed on 25 May 2018.

## 5. Conclusions

The proposed plant synthesis of silver nanoparticles using *Tanacetum vulgare* extract is an example of green nanotechnology. It is known that this type of synthesis prevents aggregation, makes it possible to modify the morphology and properties of Ag-NP surfaces, and improves their biocompatibility. The physicochemical parameters and activity of the NPs depend on the type of extract and the synthesis conditions. Due to their small size and spherical morphology, NPs show a good apoptosis rate in biomedical studies. There are many reports on the antimicrobial and anticancer activity of Ag-NPs, but objective data on the toxicity of NPs with the desired bioactivity are still needed. The published results sometimes do not indicate the activity of NPs obtained by phytogenic synthesis in relation to normal cell lines, or use a mixture from the synthesis for microbial testing, where the result is a component of the activity of the extract and trace amounts of the obtained NPs.

In our work, tests using the SNPAC, FRAP, and DPPH methods confirmed the antioxidant activity of the extracts (the highest in the case of tansy flowers), which possessed sufficient reducing power, mainly owing to presence of polyphenolic compounds, for the synthesis of Ag-NPs. The presence of hydroxyl groups next to such functional groups as ketones, aldehydes, quinones, esters, and ethers in these compounds caused the reduction of silver ions (Ag^+^) to silver nanoparticles (Ag^0^). Although the starting water extracts from *Tanaceti flos* and *Tanaceti folium* exhibited a similar composition, the *Tanaceti flos* extract contained a larger amount of amino acids and proteins, which, through carbonyl groups, bind to Ag-NPs and prevent aggregation, stabilising the dispersion in an aqueous environment. This dynamical layer on the NP surface is called a protein corona (PC) [201,202,203].

In the context of further research on the activity of the synthesised Ag-NPs, such parameters as hydrodynamic size, ζ-potential limit values, and polydispersity index, which depend on the synthesis conditions, are important. In our study, the most favourable parameters were provided by the use of water extracts applied in the ratio of 1:9 (*v*/*v*) to 1 mM AgNO_3_, which is the source of silver ions.

Regarding the anticancer activity, both aqueous extracts of *Tanaceti flos* and *Tanaceti folium* showed no significant changes in the viability of HeLa cancer cells at the tested concentrations. However, the Ag-NPs derived from *Tanaceti flos* and *Tanaceti folium* exhibited cytotoxic effects on HeLa cancer cells, with CC50 values ranging from 57.9 to 67.3 µg mL^−1^. Additionally, the Ag-NPs demonstrated a statistically significant decrease in the viability of melanoma cell lines (A375 and SK-MEL-3) at certain concentrations using the MTT assay, while the aqueous extracts did not show significant effects on the cell viability. Unfortunately, the selectivity index (SI) values were generally below 1, indicating a higher toxicity to normal cells than cancer cells. Moreover, the Ag-NPs demonstrated a statistically significant decrease also in the viability of human fibroblasts (WS-1).

The antimicrobial activity results were more promising, showing that the Ag-NPs obtained from both *Tanaceti flos* and *Tanaceti folium* exhibited strong bioactivity against various Gram-positive and Gram-negative bacteria, including antibiotic-resistant strains, with minimal inhibition concentrations ranging from 31.3 to 62.5 mg L^−1^. The Ag-NPs also showed strong activity against *Candida species* in contrast to the aqueous extracts of *Tanaceti flos* and *Tanaceti folium*, which showed weak bioactivity against the tested microorganisms.

The antimicrobial and anticancer activity of the synthesised Ag-NPs is mediated by the nanoparticle core of appropriate size and shape, the surface modification by the extract components, and the silver ions released in aqueous dispersions and biological systems. In the future, the individual components of such a hybrid can be designed to produce a formulation with the desired properties.

## Figures and Tables

**Table 1:** Total antioxidant activity of the *Tanacetum vulgare* L. extracts recalculated into equivalents.

Extract	FRAP		SNPAC		DPPH	
	µg vit.C mL^−1^ Extract	RSD%	TAC µM	RSD%	IC50 mg CGA mL^−1^ Extract	RSD%
aqueous extract of flowers	81.44	0.01	130.49	0.12	0.94	0
aqueous extract of leaves	77.25	0.01	122.31	0.13	0.94	1.08
hydroalcoholic extract of flowers	96.65	0.02	165.99	0.13	0.94	1.02
hydroalcoholic extract of leaves	85.03	0.03	140.12	0.14	0.94	1.25

Abbreviations: vit.C—ascorbic acid equivalents; TAC—the total antioxidant capacity expressed as TR equivalents; RSD%—the relative standard deviation of three independent measurements; and CGA—chlorogenic acid.

**Table 2:** Total polyphenols and flavonoids for the *Tanacum vulgare* L. extracts expressed as an equivalents of GA and Q, respectively.

Extract	Total Phenolics		Total Flavonoids	
Equivalents	µg GA mL^−1^ Extract	RSD%	µg Q mL^−1^ Extract	RSD%
aqueous extract of flowers	73.76	0.03	39.31	0.03
aqueous extract of leaves	49.43	0.01	33.84	0.02
hydroalcoholic extract of flowers	83.46	0.03	83.02	0.01
hydroalcoholic extract of leaves	80.48	0.03	49.40	0.03

Abbreviations: GA—gallic acid equivalents; Q—quercetin equivalents; and RSD%—the relative standard deviation of three independent measurements.

**Table 3:** Characteristic of Ag-NPs synthesised using aqueous extracts of flowers of *Tanacetum vulgare* L.

Parameter	Mean	Std	RSD%	Min	Max
ζ-potential (mV)	−23.30	1.09	−4.68	−22.15	−24.32
conductivity (mS/cm)	0.61	-	-	0.61	0.61
wall ζ-potential (mV)	−31.26	0.89	−2.87	−30.46	−32.23
quality factor	1.53	0.42	27.65	1.10	1.94
Z-average (nm)	88.73	0.81	0.92	88.02	89.87
polydispersity index (PDI)	0.24	0.01	4.51	0.23	0.25
mean by intensity ordered by area (nm)	114.85	2.85	2.48	111.60	118
area by intensity ordered by area (%)	100	0	0	100	100

**Table 4:** Characteristic of Ag-NPs synthesised using aqueous extracts of *Tanacetum vulgare* leaves.

Parameter	Mean	Std	RSD%	Min	Max
ζ-potential (mV)	−27.38	0.94	−3.44	−26.72	−28.46
conductivity (mS/cm)	0.94	0	0	0.94	0.94
wall ζ-potential (mV)	−27.67	1.65	−5.96	−25.82	−28.99
quality factor	2.39	0.67	27.92	1.95	3.17
Z-average (nm)	147.13	0.85	0.58	145.90	147.80
polydispersity index (PDI)	0.22	0.02	8.13	0.21	0.25
mean by intensity ordered by area (nm)	140.85	4.75	3.37	135	146.60
area by intensity ordered by area (%)	100	0	0	100	100

**Table 5:** Antimicrobial activity of the *Tanaceti flos* and *Tanaceti folium* aqueous extracts and Ag-NPs obtained in green synthesis presented as minimal inhibitory concentration (MIC) and minimal bactericidal concentration (MBC) in mg L^−1^.

Microorganism	*Tanaceti flos* Extract		*Tanaceti folium* Extract		*Tanaceti flos* Ag-NPs		*Tanaceti folium* Ag-NPs	
	MIC	MIC	MIC	MIC	MIC	MBC	MIC	MBC
Gram-positive bacteria								
*S. aureus* ATCC 25923	2000	2000	2000	2000	31.3	62.5	31.3	62.5
*S. aureus* ATCC BAA-1707 ^a^	8000	8000	4000	4000	31.3	125	31.3	125
*S. epidermidis* ATCC 12228	2000	4000	4000	4000	15.6	125	15.6	62.5
*M. luteus* ATCC 10240	8000	8000	4000	8000	7.8	125	7.8	62.5
*B. cereus* ATCC 10876	8000	>8000	8000	8000	62.5	1000	62.5	250
*E. faecalis* ATCC 29212	>8000	>8000	>8000	>8000	62.5	250	62.5	250
Gram-negative bacteria								
*S. typhimurium* ATCC 14028	>8000	Nd	>8000	Nd	31.3	31.3	31.3	62.5
*E. coli* ATCC 25922	>8000	Nd	>8000	Nd	31.3	62.5	31.3	62.5
*P. mirabilis* ATCC 12453	8000	Nd	>8000	Nd	62.5	62.5	62.5	62.5
*K. pneumoniae* ATCC 13883	>8000	Nd	>8000	Nd	31.3	125	31.3	62.5
*P. aeruginosa* ATCC 9027	>8000	Nd	>8000	Nd	15.6	31.3	15.6	31.3
Yeasts								
*C. glabrata* ATCC 90030	>8000	Nd	>8000	Nd	15.6	1000	15.6	62.5
*C. albicans* ATCC 102231	>8000	Nd	>8000	Nd	15.6	500	7.8	62.5
*C. parapsilosis* ATCC 22019	>8000	Nd	>8000	Nd	15.6	1000	7.8	1000

^a^ Methicillin-resistant *Staphylococcus aureus* (MRSA).

**Table 6:** Cytotoxicity of the *Tanaceti flos* and *Tanaceti folium* aqueous extracts and Ag-NPs obtained in green synthesis.

The Investigated Sample	Cell Line		SI
	Vero	HeLa	
	CC_50_ (µg mL^−1^) ^a^		
*Tanaceti flos* extract	596.6 ± 6.0	681.0 ± 6.0	0.9
*Tanaceti folium* extract	861.3 ± 102.5	753.1 ± 46.3	1.1
*Tanaceti flos* Ag-NPs	22.1 ± 5.4	67.3 ± 0.2	0.3
*Tanaceti folium* Ag-NPs	14.1 ± 0.3	57.9 ± 3.3	0.2

Abbreviations: The CC_50_ is the cytotoxic concentration required to reduce the number of viable cells by 50%; SI = CC_50_ of the tested extract in normal Vero cell line/CC_50_ of the tested extract in a cancer cell line; ^a^ mean ± S.D. values come from two independent experiments.

**Table 7:** The IC50 values * expressed in µg mL^−1^.

The Investigated Sample	SK-MEL-3 Cell Line	A375 Cell Line	WS1 Cell Line
*Tanaceti flos* extract	>100	>100	>100
*Tanaceti folium* extract	>100	>100	>100
*Tanaceti flos* Ag-NPs	14.53	22.04	4.83
*Tanaceti folium* Ag-NPs	18.57	34.98	4.87

* The IC50 values were calculated using an online IC50 calculator: https://www.aatbio.com/tools/ic50-calculator, accessed on 25 May 2018.

## Data Availability

The data presented in this study are available on request from the corresponding authors.

## Sample Availability

Compound samples are available from the authors.
